# Management and Outcomes of Umbilical Hernia in Decompensated Chronic Liver Disease: A Single-Unit Experience of Six Cases

**DOI:** 10.7759/cureus.82578

**Published:** 2025-04-19

**Authors:** Karamveer Singh, Manoj Joshua Lokavarapu, Nayana S Kumar, Amit Gupta, Arunkumar V, Dipendra Singh, Vatsala Singh, Mohammad Shahid Raja, Satish Ammapalem, Monisha Selvarasu

**Affiliations:** 1 General Surgery and Division of Organ Transplant, All India Institute of Medical Sciences, Rishikesh, Rishikesh, IND; 2 General Surgery, All India Institute of Medical Sciences, Rishikesh, Rishikesh, IND; 3 General Surgery, All India Institute of Medical Sciences, Rishikesh, rishikesh, IND

**Keywords:** decompensated chronic liver disease, emergency hernia repair, end-stage liver disease, transjugular intrahepatic portosystemic shunt, umbilical hernia

## Abstract

Background

Complicated umbilical hernia in decompensated chronic liver disease is a significant cause of morbidity and mortality. This study evaluates the management strategies and factors predicting outcomes following emergency umbilical hernia repair.

Methods

A retrospective analysis was conducted on six patients with decompensated chronic liver disease who presented with complicated umbilical hernia in an emergency setting to the hepatopancreatobiliary (HPB) surgical unit between July and December 2024. Preoperative characteristics, intraoperative findings, and postoperative outcomes were reviewed.

Results

A total of six patients were included. The mean age was 51.16 years (range: 44-58), and all presented with abdominal pain (6, 100%). Among them, two (33.3%) presented with a strangulated umbilical hernia, two (33.3%) with an obstructed hernia, and two (33.3%) with a ruptured hernia associated with ascites fluid leak. All patients (6, 100%), underwent emergency surgery. Bowel resection was required in three (50.0%) patients, of whom two (33.3%) underwent primary anastomosis and one (16.7%) underwent a double-barrelled ileostomy. All six (100%) patients underwent primary repair of the hernial defect without mesh placement. Postoperative recovery was uneventful in four (66.7%) patients; one (16.7%) patient developed an ascites fluid leak, and one (16.7%) died. The mean postoperative hospital stay was 14.83 days.

Conclusion

Emergency repair of complicated umbilical hernia in patients with decompensated chronic liver disease is associated with high morbidity and mortality. Contributing factors include increased ascites, portal hypertension, and liver decompensation following surgery. Preoperative optimization and intraoperative ascites drainage may play a crucial role in improving outcomes, even in emergency settings.

## Introduction

Umbilical hernias occur in up to 20-40% of patients with decompensated chronic liver disease (DCLD) [1]. Increased intra-abdominal pressure due to ascites and sarcopenia are the main contributing factors, and if not treated, it may present as complications like incarceration, strangulation, skin necrosis, leaking ascites, spontaneous bacterial peritonitis, and hernia rupture with evisceration [2-4].

Management of umbilical hernia in decompensated chronic liver disease is not standardised. Patients with decompensated chronic liver disease have a significantly higher morbidity and mortality during elective hernia repair. These risks are even more when it is an emergency surgery. Recent literature encourages elective repair to avoid the added risk of emergency surgery [5]. Alternatively, a proportion of patients may be eligible for liver transplantation, and deferring repair to the time of transplantation may be preferred. However, predicting the timing of transplantation limits the effectiveness of this approach [6,7].

Postoperative morbidity and mortality in patients with decompensated liver disease are attributed to the degree of portal hypertension and decompensation of liver function following surgery [5,8]. So, we have to reduce the portosystemic pressure gradient and ascites, which is a common postoperative complication following surgery and a particular problem in patients requiring umbilical hernia repair, as it may lead to breakdown of the surgical wound following repair. The use of transjugular intrahepatic portosystemic shunt (TIPS) to decompress portal pressure preoperatively is advocated [9,10].

## Materials and methods

Study design and setting

This retrospective study was conducted in the hepatopancreaticobiliary (HPB) unit of the Department of Surgery at the All India Institute of Medical Sciences, Rishikesh, India. We analyzed all patients who underwent emergency umbilical hernia repair in the setting of decompensated chronic liver disease between July 2024 and December 2024.

Inclusion and exclusion criteria

We included patients aged 18 years or older who underwent emergency surgical repair for complicated umbilical hernia in the setting of decompensated chronic liver disease during the study period. Due to the study’s retrospective nature, no specific exclusion criteria were applied to ensure a complete dataset of all patients managed in our unit.

Data collection

The Institutional Ethics Committee of the All India Institute of Medical Sciences, Rishikesh, approved the study (AIIMS/RISHI/GS/2025-246) prior to data collection. Patient data were retrieved from medical records, operative notes, and electronic hospital databases. The following variables were collected and analyzed.

Preoperative Characteristics

These included demographic data (age, gender), clinical presentation, severity of decompensation (ascites, hepatic encephalopathy, coagulopathy), and any preoperative imaging findings.

Biochemical Parameters

Liver function tests (serum bilirubin, alanine aminotransferase, aspartate aminotransferase, albumin, and international normalized ratio), renal function tests (serum creatinine and blood urea nitrogen), and complete blood counts were recorded.

Intraoperative Findings

These included the presence of bowel strangulation or perforation, peritoneal contamination, hernia sac contents, and the presence of ascites intraoperatively.

Surgical Details

The type of surgical approach (primary repair vs. mesh repair), intraoperative techniques used to manage ascites, and any adjunctive procedures performed were documented.

Postoperative Outcomes

Postoperative clinical outcomes included the duration of hospital stay, development of postoperative complications such as surgical site infections, ascitic leaks, ileus, hepatic decompensation, and reoperation rates. In-hospital mortality and any need for readmission were also recorded.

Statistical analysis

The statistical analysis was conducted using SPSS version 21.0 (IBM Corp., Armonk, NY, USA). Descriptive statistics were used to summarize patient demographics, intraoperative findings, and postoperative outcomes. Categorical variables were presented as frequencies and percentages, while continuous variables were summarized as mean ± standard deviation (SD).

## Results

Six patients were included in the study. Table 1 summarizes the preoperative characteristics of individuals who underwent umbilical hernia repair. The mean age was 51.16 years (range, 44-58 years), with a male-to-female ratio of 5:1. Abdominal pain was the most common presenting symptom in all patients ( 100%). Out of six patients, two (33.33%) had strangulated umbilical hernia, two (33.33%) presented as obstructed umbilical hernia, and two (33.33%) as ruptured umbilical hernia with ascites fluid leak. All six patients were optimised preoperatively with diuretics, paracentesis, albumin transfusions, and fresh frozen plasma (FFP) transfusions, and the Child-Turcotte-Pugh (CTP) score and Sodium Model for End-Stage Liver Disease (Na MELD) score were calculated; all six underwent emergency surgery. Three (50%) patients underwent resection of the gangrenous segment of bowel and two (33.33%) had primary bowel anastomosis and one (16.66%) patient underwent double barrel ileostomy. We had two (33.33%) patients in Child class C and four (66.66%) patients in Child class B. The mean Na MELD score of the cohort was 23.16 ± 3.99. The median score was 23.5, with a range of 19 to 28. All six (100%) patients underwent primary repair of umbilical hernia without mesh placement. An intraoperative drainage tube was placed in all six patients (100%), allowing controlled postoperative drainage of ascitic fluid, with an average duration of retention exceeding 14 days. Out of the six patients, four (66.66%) patients had uneventful postoperative recovery, but one (16%) patient developed ascites leak from the wound site, and one (16.7%) patient expired in the postoperative period. Mean postoperative stay for the patients was 14.83 days (Table 2).

**Table 1:** Preoperative characteristics of individuals who underwent umbilical hernia repair. DM: Diabetes mellitus, SGOT: serum glutamic oxaloacetic transaminase, SGPT: serum glutamic pyruvic transaminase, Na: Sodium, K: Potassium, Ca: calcium, PT: Prothrombin time, INR: international normalised ratio, HCV: Hepatitis C virus, PTB: Pulmonary tuberculosis, DCLD: Decompensated chronic liver disease, Na MELD: Sodium Model for End-Stage Liver Disease.

Characters	Patient 1	Patient 2	Patient 3	Patient 4	Patient 5	Patient 6
Age and sex	44/M	58/F	52/M	64/M	44/M	45/M
Symptoms	Abdominal pain, vomiting, obstipation	Abdominal pain, leaking ascites through ruptured umbilical hernia	Abdominal Pain, leaking ascites through rupture umbilical hernia.	Abdominal Pain, obstipation	Abdominal Pain	Abdominal pain, fever, vomiting
Signs	Diffuse tenderness, strangulated umbilical hernia	Diffuse tenderness,ruptured umbilical hernia	Ruptured umbilical hernia	Diffuse tenderness, strangulated umbilical hernia	Diffuse tenderness with obstructed umbilical hernia	Diffuse tenderness, obstructed umbilical hernia
Comorbidities	DM DCLD	HCV Positive, DCLD	DM, DCLD	DCLD	DCLD	DCLD
Hemoglobin (Normal range: 13-17g/dL)	7.7	11	10	8.6	7.6	8.9
Total count (Normal range: 4,000-11,000 cells/ µL)	14100	8420	14530	7730	6390	11800
Platelet (Normal range:150000-400000/ µL)	56000	120000	100000	78000	130000	210000
Total bilirubin (normal range: 0.3-1.2 mg/dl)	7.6	2.36	12.9	0.98	0.39	1.09
Direct bilirubin (normal range: 0-0.2 mg/dl)	3.12	1.32	7.08	0.1	0.16	0.65
SGOT (normal range: 0-35 U/L)	50	28	53	31	32	62
SGPT (normal tange: 0-35 U/L)	64	52	59	50	33	77
Alkaline phosphatase (normal range: 30-120 U/L)	140	113	390	139	181	189
Albumin (3.5-5.2g/dL)	2.6	2.4	2.8	2.1	2	2.4
Serum Sodium (Normal Range: 135-146 mmol/L)	138	134	130	130	128	126
Serum Potassium (Normal Range: 3.5-5.1 mmol/L)	4	4.5	4	4	4.3	4.3
Serum calcium (Normal Range:101-109 mmol/L)	8	7.3	8.2	8.7	7.1	8
Blood Urea (Normal Range: 17-43 mg/dL)	67	33	34	35	63	24
Serum creatinine (Normal Range:0.72-1.18 mg/dL)	1.13	0.88	0.9	1.13	1.42	0.48
PT/INR	31.4/3.0	18.6/1.63	19.6/1.63	16.4/1.44	15.6/1.24	30/2.76
Child Turcotte Pugh score	Class C	Class B	Class B	Class B	Class B	Class C
Na MELD	28	19	26	19	21	26

**Table 2:** Illustrates intraoperative findings, procedure done and duration of hospital stay and postoperative complications. MODS: multiple organ dysfunction syndrome.

No.	Age/sex	Preoperative diagnosis	Intra-op findings	Surgery	Duration of hospital stay	Post operative complications
1	44/M	Strangulated umbilical hernia	15 cm gangrenous ileal segment 3O cm from ileocaecal junction	Resection of ileal segment with double barrel ileostomy, primary repair of umbilical hernia with umbilectomy	30 Days	Ascites leak
2	58/F	Ruptured umbilical hernia with ascites leak	2x2cm umbilical hernia defect with congested omentum as content.	Resection of the omentum with primary repair of hernia with umbilectomy	14	Nil
3	52/M	Ruptured umbilical hernia with ascites leak	1x 1.5 cm umbilical hernia defect with omentum as content.	Resection of omentum with primary repair of umbilical hernia.	10	Nil
4	64/M	Strangulated umbilical hernia	20cm gangrenous ileal segment 50 cm proximal to ileocaecal junction	Resection of gangrenous ileal segment and ileo-ileal side to side anastomosis, with primary repair of umbilical hernia with umbilectomy	11	Nil
5	44/M	Obstructed umbilical hernia	Gangrenous ileal segment 50 cm from Ileocaecal junction	Resection of ileum with ileo-ileal anastomosis, primary repair of umbilical hernia with umbilectomy	12	Nil
6	45/M	Obstructed umbilical hernia	1x 0.5 cm defect with bowel and omentum as content.	Primary repair and umbilectomy	12	MODS, death

Exemplary case presentations

Patient 1

**Figure 1:**
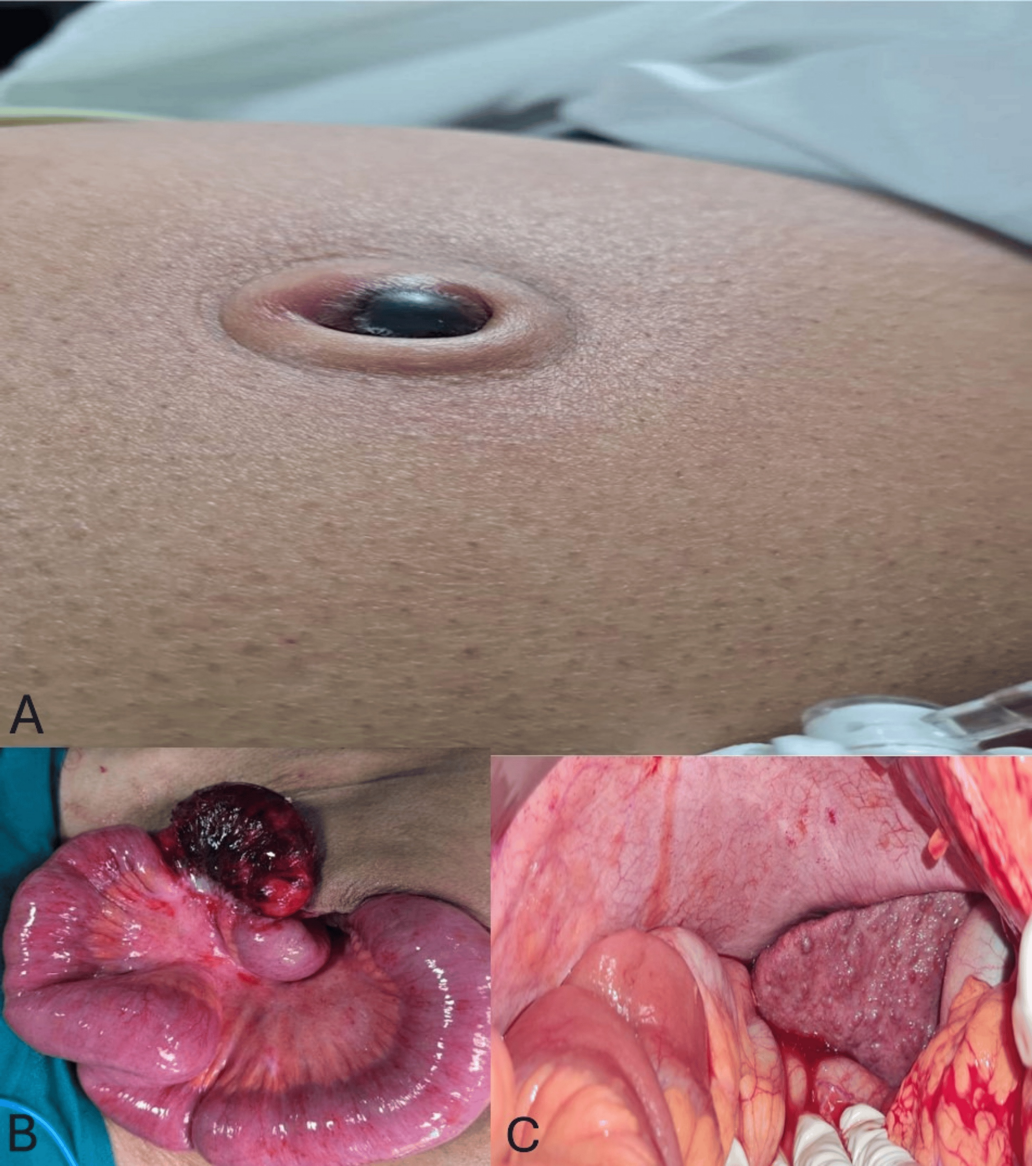
Clinical and Intraoperative Findings in a Patient with Strangulated Umbilical Hernia with Decompensated Chronic Liver Disease. (A) Clinical photograph of Patient 1 presenting with a strangulated umbilical hernia, showing overlying skin changes indicative of compromised perfusion. (B) Intraoperative photograph of Patient 1 demonstrating a gangrenous segment of the ileum, necessitating resection. (C) Intraoperative photograph of Patient 1 revealing a cirrhotic liver with a nodular surface and altered texture, consistent with chronic liver disease. Picture credits: Ammapalem Satish

A 44-year-old male with a known case of decompensated chronic liver disease with portal hypertension presented with complaints of abdominal pain, vomiting, and obstipation for three days, diagnosed as a strangulated umbilical hernia. Preoperative optimization with albumin and fresh-frozen plasma transfusions was performed. He underwent emergency exploratory laparotomy and intraoperatively found to have a 15 cm gangrenous ileal segment, 30 cm proximal to an ileocaecal junction (Figure 1). He underwent resection of the ileal segment with double-barrelled ileostomy with primary repair of the umbilical hernia with umbilicotomy. He developed ascites leak from the incision site on postoperative day six, which was managed with sodium restriction, diuretics, controlled drainage through the intra-abdominal drain, and albumin transfusions. The patient was discharged on postoperative day 30, with no other complications.

Patient 3

**Figure 2:**
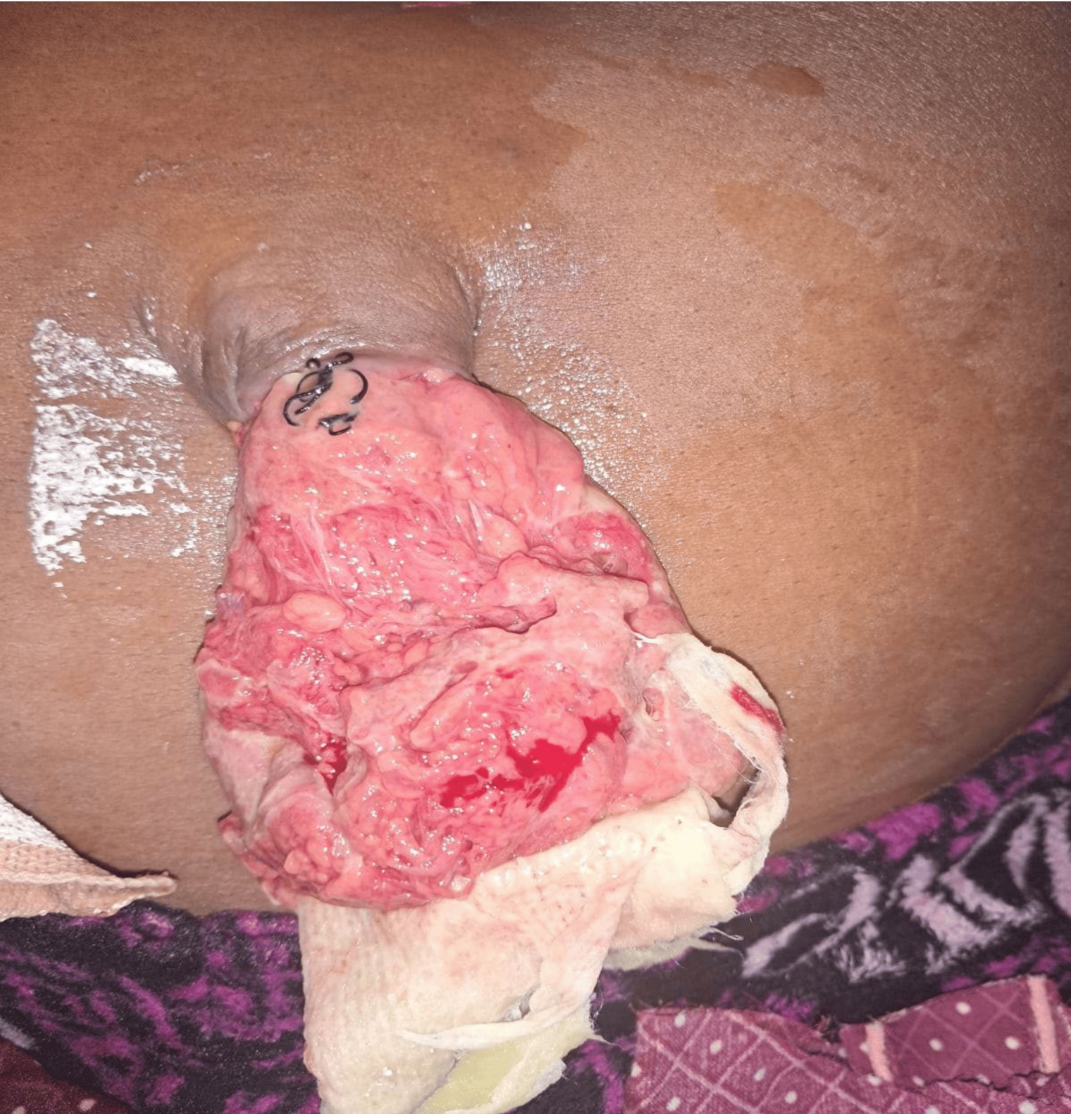
Clinical photograph of Patient 3 showing an umbilical hernia with evisceration of omentum through a ruptured hernial sac Picture credits: Ammapalem Satish

A 52-year-old male patient, a known case of decompensated chronic liver disease with portal hypertension and diabetes mellitus, presented with complaints of abdominal pain and ruptured umbilical hernia with leaking ascites and evisceration of omentum (Figure 2). He underwent emergency exploratory laparotomy and resection of the herniated omentum with primary repair of the umbilical hernia with umbilectomy. His postoperative period was uneventful, and the intrabdominal drain was removed on postoperative day eight. He was discharged on postoperative day 10.

## Discussion

Umbilical hernia management in patients with DCLD is a topic of debate. Determining the indication, timing, and type of surgery to be performed is challenging. The morbidity and mortality, recurrence following surgery in DCLD patients, make the surgeons think twice before planning for surgery because of these factors, a wait-and-watch policy has been advocated by even high-volume centres. In our case series, all patients presented with either bowel obstruction, strangulation, or ruptured umbilical hernia, necessitating emergency surgical intervention in every case (Table 2).

Umbilical hernia is noted to be as high as 20-40% in patients with DCLD with refractory ascites, compared to the general population, where the incidence is only 2-3% [1,2]. The etiology of umbilical hernia is multifactorial, cirrhosis related sarcopenia associated fascial weakness, poor nutritional status, portal hypertension and variceal formation causes venous dilatation and recanalization of umbilical vein and rapid fluid accumulation, increase in intrabdominal pressure is the reason for necrosis of the overlying skin leading to spontaneous rupture, peritonitis and evisceration [3,4,6].

The main risk factors for poor outcome are the degree of portal hypertension, underlying organ dysfunction, and ascites. Studies show that the patient's result can be improved if the factors above are addressed preoperatively. The mortality risk scores used are the CTP score, a MELD score, and the Na MELD score, which is especially useful in surgical candidates with DCLD. CTP class A or MELD < 10: elective surgery is considered. CTP class B/C or MELD > 10: optimize the patient with medical therapy and ensure adequate perioperative ascites control. More specifically, in patients with MELD scores above 15 or CTP class C, the perioperative morbidity and mortality are exceptionally high, so simultaneous liver transplantation and hernia repair should be considered [8,11,12].

Management of ascites in DCLD patients with umbilical hernia plays a very important role in outcome, and it should be done initially with diuretic therapy and a sodium-restricted diet. However, in cases of refractory ascites, intermittent paracentesis followed by albumin administration and/or TIPS should be considered [13,14].

Placement of abdominal drains preoperatively or intraoperatively to divert ascites away from the operative site, or a peritoneovenous (PV) shunt or surgical portosystemic shunt, has been extensively used in the past to reduce ascites reaccumulation [15-17,9].

But despite all the above-mentioned measures, TIPS has proven to be safer and more effective by various randomized controlled trials in managing ascites, improving transplant-free survival, treating/preventing variceal bleeding, and reducing postoperative mortality and morbidity in the presence of portal hypertension. However, these advantages have to be weighed against the risk of inducing hepatic encephalopathy, ischemic hepatopathy, and possibly complicating subsequent liver transplantation [10,18].

A conservative wait-and-watch approach in patients with poorly optimized conditions leads to complications and recurrences [19]. In the current setting, there is more evidence to support elective hernia repair over emergency surgery. The potential complications of the wait-and-watch policy include rupture, evisceration, ascites fluid leak, peritonitis, strangulation, obstruction, and emergency surgeries, which are associated with poor patient outcome, recurrence, and prolonged hospital stay [20]. After optimizing ascites and other factors, all patients should be considered for elective surgery, including those with complicated umbilical hernias. A semi-elective repair should be encouraged after ascites control, which is a significant factor in reducing morbidity and mortality [21].

To determine whether to proceed with a laparoscopic or open approach, several studies have demonstrated that patients undergoing laparoscopic repair experience superior outcomes, characterized by low postoperative morbidity and mortality, as well as shorter recovery times and hospital stays [22,23]. Regarding mesh versus primary repair, the current literature suggests that the use of a mesh in elective hernia repair for patients with cirrhosis is a safe alternative, with lower recurrence rates compared to primary suture [24-26].

DCLD patients with umbilical hernia undergoing liver transplantation should be encouraged to have hernia repair during the same procedure, as a persistent hernia in the post-transplant period carries a higher risk of hernia-related complications, and it can be done through the same incision used for transplant [6].

In a specific subset of patients with umbilical vein recanalization and severe portal hypertension, acute portal vein thrombosis followed by liver failure has been reported after umbilical hernia repair, so a conservative approach should be followed in such patients until liver transplantation [7,27].

Limitations

This retrospective study has several limitations. The small sample size (six patients) limits the generalizability of findings, necessitating larger studies for more robust evidence on umbilical hernia management in DCLD. The absence of a control group prevents direct comparisons between emergency and elective surgery or different management strategies (e.g., TIPS vs. no TIPS), limiting definitive conclusions on the superiority of any approach. Variability in preoperative optimization (e.g., albumin and FFP transfusions, paracentesis) may introduce bias, as liver function and ascites control were not uniform across cases. Given the complexity of DCLD with portal hypertension, extended follow-up is crucial to assess the long-term efficacy and safety of hernia repair. More extensive prospective studies with control groups and long-term follow-up are needed to validate these findings and optimize management strategies.

## Conclusions

Emergency umbilical hernia repair in patients with decompensated chronic liver disease remains a high-risk procedure, often necessitated by life-threatening complications. Optimizing ascites control and liver function before surgery is crucial in improving outcomes. While elective repair is increasingly favored over a conservative approach, individualized management based on disease severity is essential. The role of TIPS in preoperative optimization, as well as the choice between mesh and primary repair, requires further evaluation. Larger prospective studies are needed to establish standardized guidelines for the optimal timing and approach to umbilical hernia repair in this high-risk population.
